# How fragile the positive results of Chinese herbal medicine randomized controlled trials on irritable bowel syndrome are?

**DOI:** 10.1186/s12906-024-04561-8

**Published:** 2024-08-14

**Authors:** Minjing Luo, Jinghan Huang, Yingqiao Wang, Yilin Li, Zhihan Liu, Meijun Liu, Yunci Tao, Rui Cao, Qianyun Chai, Jianping Liu, Yutong Fei

**Affiliations:** 1 Centre for Evidence-Based Chinese Medicine, Beijing University of Chinese Medicine, No.11, Bei San Huan Dong Lu, Chaoyang District, Beijing, 100029 China; 2 School of Traditional Chinese Medicine, Beijing University of Chinese Medicine, Beijing, 100029 China; 3 School of Qi-Huang Chinese Medicine, Beijing University of Chinese Medicine, Beijing, 100029 China

**Keywords:** Fragility index, Randomized controlled trial, Chinese herbal medicine, Irritable bowel syndrome, Research methodology

## Abstract

**Objective:**

The fragility index (FI), which is the minimum number of changes in status from “event” to “non-event” resulting in a loss of statistical significance, serves as a significant supplementary indicator for clinical physicians in interpreting clinical trial results and aids in understanding the outcomes of randomized controlled trials (RCTs). In this systematic literature survey, we evaluated the FI for RCTs evaluating Chinese herbal medicine (CHM) for irritable bowel syndrome (IBS), and explored potential associations between study characteristics and the robustness of RCTs.

**Methods:**

A comprehensive search was conducted in four databases in Chinese and four databases in English from their inception to January 1, 2023. RCTs encompassed 1:1 ratio into two parallel arms and reported at least one binary outcome that demonstrated statistical significance were included. FI was calculated by the iterative reduction of a target outcome event in the treatment group and concomitant subtraction of a non-target event from that group, until positive significance (defined as *P* < 0.05 by Fisher’s exact test) is lost. The lower the FI (minimum 1) of a trial outcome, the more fragile the positive result of the outcome was. Linear regression models were adopted to explore influence factors of the value of FI.

**Results:**

A total of 30 trials from 2 4118 potentially relevant citations were finally included. The median FI of total trials included was 1.5 (interquartile range [IQR], 1–5), and half of the trials (*n* = 15) had a FI equal to 1. In 12 trials (40%), the total number of participants lost to follow-up surpassed the respective FI. The study also identified that increased FI was significantly associated with no TCM syndrome differentiation for inclusion criteria of the patients, larger total sample size, low risk of bias, and larger numbers of events.

**Conclusions:**

The majority of CHM IBS RCTs with positive results were found to be fragile. Ensuring adequate sample size, scientifically rigorous study design, proper control of confounding factors, and a quality control calibration for consistency of TCM diagnostic results among clinicians should be addressed to increase the robustness of the RCTs. We recommend reporting the FI as one of the components of sensitivity analysis in future RCTs to facilitate the assessment of the fragility of trials.

**Supplementary Information:**

The online version contains supplementary material available at 10.1186/s12906-024-04561-8.

## Introduction

Hypothesis testing is fundamental in statistical analysis, aiding in discerning significant differences between experimental samples or populations [1, 2]. In randomized controlled trials (RCTs), hypothesis testing using p-value as the probability value is used as an indispensable and extensively utilized tool to draw significant statistical conclusions when p-value is smaller than the predefined significance level “α” [3–7].

The fragility index (FI) concept is significant, as it measures the minimum number of events required for an outcome to shift from statistically significant to nonsignificant [8]. A lower FI value indicates a more fragile result, and suggests that the statistical significance of the outcome is sensitive to small changes. Besides, FI also can indicate a fragile result when encountering trials with limited clinical events or small-scale studies [9], since their results may be misleading.

Introduced in 2014 by Professor Walsh and his team [8], the FI has been investigated across various medical fields, such as spine surgery [10], hand surgery [11], critical care [12], and anti-cancer drugs [13]. FI holds paramount importance in assessing the robustness of clinical trials. Its primary objective is to provide fragility insights to patients, clinicians, and policymakers, enabling a comprehensive understanding of clinical trial results and facilitating well-informed clinical decision-making [14].

Traditional Chinese medicine (TCM) faces unique challenges when evaluating trial result fragility. It adopts individualized and complex treatments in response to the TCM syndrome [15, 16], which is a combination of multiple symptoms and signs subjectively observed and organized [17–19]. The subjectiveness of TCM syndrome differentiation induces incoherence among practitioners while practice, and thus may put the results of clinical trials of TCM in a higher risk of being fragile.

Our objective is to assess the FI in RCTs comparing Chinese herbal medicine (CHM) treatments for irritable bowel syndrome (IBS). We aim to explore potential associations between study characteristics and the robustness of the RCTs, illustrating the fragility of positive results and underscoring the importance of reporting FI for a more accurate and scientific understanding of positive CHM trial results.

## Methods

### Literature search

A search of Medline (Ovid), Embase, Cochrane Library, Web of Science, China National Knowledge Infrastructure (CNKI), SinoMed, China Science and Technology Journal database (VIP), and Wan-Fang database from their inception until January 1, 2023 was conducted to identify potentially eligible studies. The search strategy was structured using “Chinese herbal medicine” “irritable bowel syndrome”, and “randomized controlled trial” with no language restrictions were imposed (Supplementary Table S1). In this study, we included only published RCTs on the treatment of CHM for IBS. The reference lists of all included studies and relevant systematic reviews were checked for further reports and contacted trial authors where necessary. In conformance with the Preferred Reporting Items for Systematic Reviews and Meta-analyses (PRISMA) guidelines [20] (Supplementary Table S2), all supporting data have been provided in the article and the supplementary data.

### Study selection

#### Types of trials

We included RCTs that utilized a 1:1 ratio for treatment allocation and reported binary outcomes with statistical significance. In order to maintain the integrity of the analysis, trials that employed inappropriate methods of randomization were excluded from the study. The criteria used to determine the appropriateness of randomization method was defined as an appropriate detailed description of generation methods for random sequences. Randomization that was described only by the word “random” or was conducted via assigning patients to different groups by time of admission, date of birth, or number of hospital medical records and so on was defined inappropriate.

#### Types of participants

Trials were conducted among patients afflicted with IBS. There are no restrictions on the subtypes, including diarrhea-predominant, constipation-predominant, mixed, or other types.

#### Types of interventions

The included interventions encompassed three main categories: (1) single herb; (2) Chinese proprietary herbal medicine, typically administered as granules, decoction, oral liquid, capsule, or pills; (3) herbal compound decoction prescribed by TCM doctors, that discriminated based on the specific symptoms and conditions exhibited by the patients. The study did not impose any restrictions on the formulation or integration of different herbal medicines. However, studies involving non-oral administration modes for herbal medicines were excluded from the analysis.

#### Comparison group

The control group in the included studies consisted of various interventions, including: (1) placebo; (2) standard treatment, interventions recommended by clinical guidelines; (3) treatment as usual, the routine medical care provided to patients in the study group; (4) another alternative oral CHM; (5) integrative medicine, interventions combining CHM with standard treatment, routine treatment or an alternative oral CHM; and (6) no treatment.

#### Outcomes

Trials included in this study reported at least one statistically significant binary outcome in their abstracts. We included effective rate [21] and the response rate [22] of the IBS Symptom Severity Scale (IBS-SSS), adequate relief (IBS-AR) [23], response rate of abdominal pain (visual analogue scale, VAS scale) [24], and response rate on Bristol Stool Scale [25]. Based on the IBS-SSS scale, there were 4 graded outcomes including remission (less than 75 points), mild (76–175 points), moderate (176–300 points), and severe (over 300 points), respectively. Remission was considered as cured, 2 grades improvement as markedly improved, 1 grade improvement as improved, no improvement or worsen condition as ineffective. Effective rate [21]= (cured + markedly improved + improved) / total cases × 100%. Additionally, the response rate [22] on the IBS-SSS scale was defined as the proportion of patients who had a ≥ 50% reduction in the total score compared to the pre-treatment. IBS-AR [23] was defined as a binary answer (Yes/No) to the question “In the past 7 days, have you had adequate relief of your IBS symptoms?” Response rate to abdominal pain [24] was defined as the proportion of patients whose worst abdominal pain score (score range, 0–10, with 0 indicating no pain and 10 indicating unbearable severe pain) decreased by at least 30%, and response rate on Bristol Stool Scale was defined as the proportion of patients whose type 6 or 7 stool days decreased by 50% or greater [25].

### Data collection process

One review author (Li YL) extracted data from 2 included trials into drafted, piloted, Excel-based extraction forms, and a second review author (Wang YQ) checked data entry in full to pilot the electronic data extraction form. Four reviewers (Li YL, Wang YQ, Huang JH, and Liu ZH) independently extracted data from the eligible studies into the formal form. Discrepancies were resolved through discussion, or if needed, through consultation with another overview author (Luo MJ). In order to ensure the consistency in data extraction, we conducted an example data extraction training using one of included RCT reports priory to the formal. The information extracted for each eligible RCT included the data related to the target outcome, such as the number of events and nonevents for each group. Furthermore, the following data were also extracted: the basic characteristics of patients (gender, mean age), the pathological type of IBS, duration of the condition, the TCM syndrome differentiation and typing of IBS (yes or unclear), the flexibility of interventions (whether the interventions were tailored to the patient’s condition, yes or unclear), the type of interventions comparisons (placebo, standard treatment or treatment as usual), total sample size, number of patients lost to follow-up, year of publication, funding status, adequacy of allocation concealment (recorded as adequate or unclear), patient and investigator blinding, and statistical analysis principle. In cases where more than one significant binary outcome was reported in the abstract of a trial, only the primary outcome was included in the assessment of the FI.

### Risk of bias assessment

Two reviewers who have good calibration of the Cochrane Collaboration’s risk of bias tool 2.0 (RoB 2.0) [26] independently assessed the risk of bias for each included RCT. RoB 2.0 is consisted of six domains including randomization process, deviations from intended interventions, missing outcome data, measurement of the outcome and selection of the reported result. Each domain contained several signal questions were judged with five potential responses: ‘Yes’, ‘Probably Yes’ ‘Probably No’, ‘No’ and ‘No Information’. In all cases, a judgement of ‘Yes’ indicated a low risk of bias, a judgement of ‘Probably Yes’ or ‘Probably No’ indicated some concerns and a judgement of ‘No’ indicated a high risk of bias. If insufficient detail was reported, our judgement would be ‘No Information’. We resolved disagreements that arise at any stage by discussion between the review authors or with a third reviewer, when necessary. We assessed the domains above using answers to signaling questions and generated a risk of bias’s assessment table for each study, with overall judgments derived from the tool.

### Statistical analysis

**Fig. 1:**
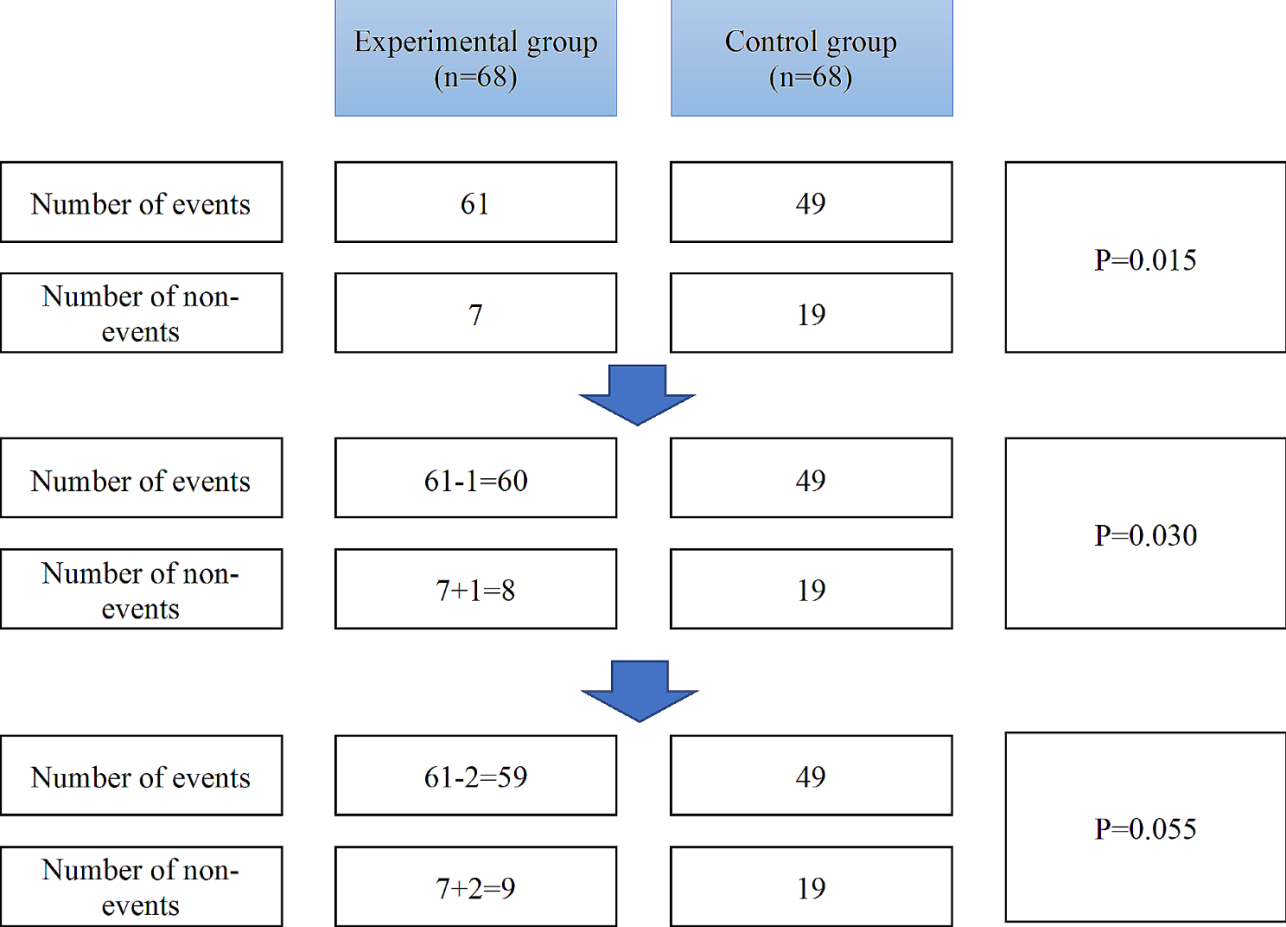
Example of fragility index calculation for the trial titled “Shugan Liqi Zhixie Tang in Treatment of 68 Patients with Diarrhea-predominant irritable bowel syndrome”

For the included binary outcome, the FI was calculated from a two-by-two contingency table by incrementally changing 1 patient at a time from an “event” status to a “non-event” status in the treatment group [8]. This process was executed in a way that maintained the total number of participants in that treatment. After each change, the Fisher exact test was recalculated, and the resulting two-sided *P* value was recorded. This iterative process continued until the *P* value reached ≥ 0.05, indicating that the result was no longer statistically significant (see Fig. 1 for calculation example). The minimized required number of subtracted target outcome event occurrences was considered the FI for that RCT outcome. This index serves as a valuable measure to evaluate the fragility of the study results, reflecting the sensitivity of the statistical significance to increased number of events, and at the same time, to manifest the robustness of the results.

We summarized the FI for the included studies using descriptive statistics and described the distribution of FI frequency using histogram. A linear regression model was conducted to explore potential factors that might have an influence on fragility index. As the FI was highly skewed, it was log transformed and the categorical variables were transferred into dummy variables prior to the regression analysis. The assumptions underlying linear regression were examined and confirmed [27]. Based on the previous studies related to the exploration of fragility index [8, 28, 29], potential factors that influenced the fragility index including total sample size, the type of interventions comparison (placebo, standard treatment, or treatment as usual), the proportion of patients lost to follow-up, the total number of events in the trial, the risk of bias assessment, and funding status. In addition, we pay special attention to the characteristics of TCM intervention. According to TCM theory, most TCM experts typically implement syndrome-based TCM treatments using various forms of TCM formulas, appropriately combined with individual herbs that differ in nature and medical value. However, due to the subjectivity of diagnosing patients’ syndromes and the inherent flexibility in intervention measures, the robustness of clinical trial results may be affected. We reported the regression coefficients and the 95% confidence intervals (CIs), obtained using 1000 bootstrap samples, providing a robust statistical analysis approach.

## Results

**Fig. 2:**
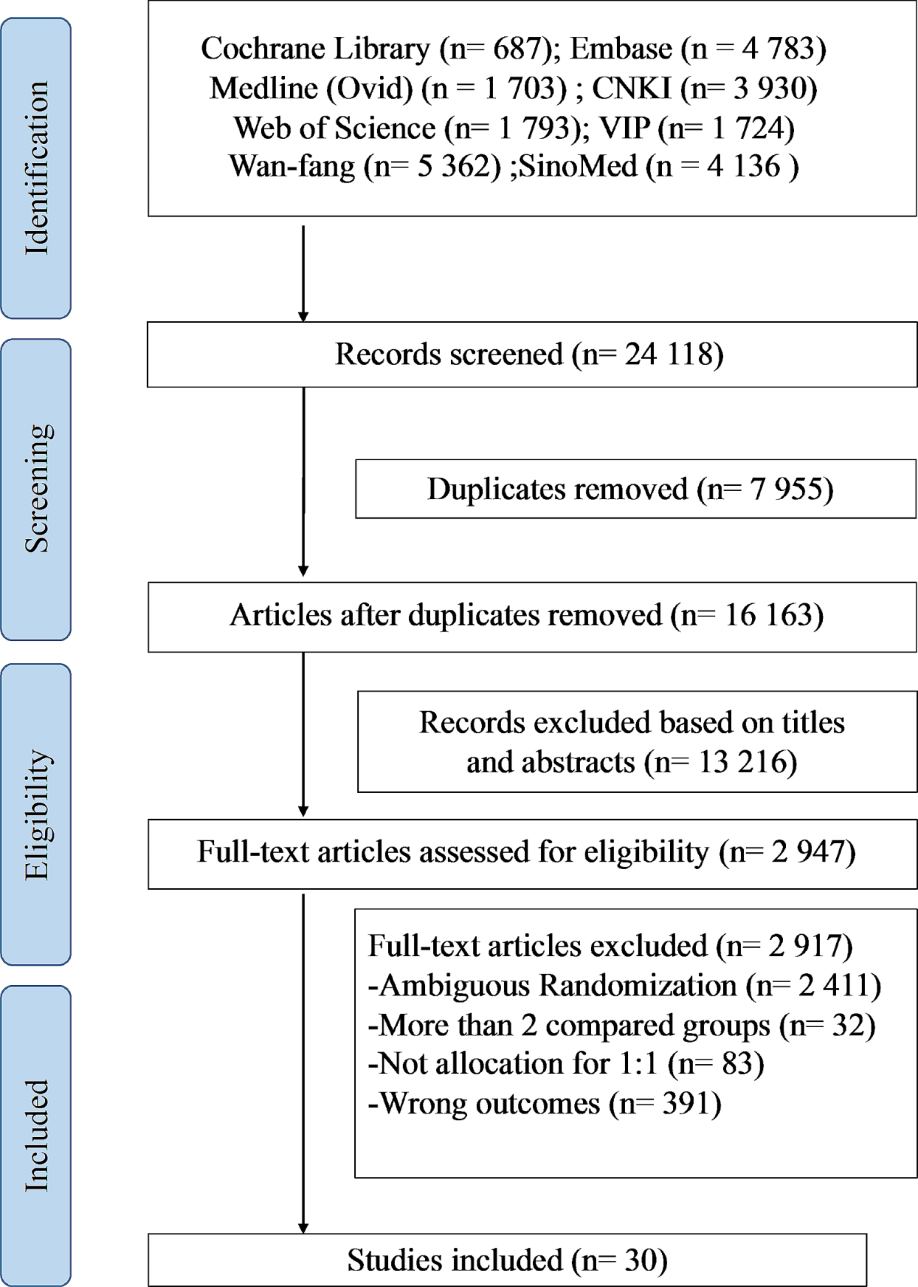
Details of the literature search

The initial search yielded a total of 24 118 potentially relevant citations. Thirty RCTs that met the inclusion criteria and were eligible for analysis (Fig. 2).

### Characteristics of trials and outcomes

Table 1 shows the comprehensive data on the specific outcomes extracted from each study. The median sample size of the included studies was 72 patients (range, 50–360), with a median of 4 patients (range, 1–14) who were lost to follow-up. Regarding the reported outcomes, 18 studies (60%) focused on the effective rate, 4 studies (13.33%) examined the response rate on the IBS-SSS Scale, while 3 studies (10%) assessed the response rate to abdominal pain, 2 studies (6.67%) reported data on adequate relief and another 3 studies (10%) measured the response rate on the Bristol Stool Scale. In terms of the utilization of TCM in the trials, approximately two-third (70%) of the eligible trials incorporated patients’ TCM syndromes as criteria for inclusion. However, only one in six of the trials’ interventions (16.67%) were flexibly tailored to the patient’s condition based on TCM theory. In the 18 trials that clearly reported lost to follow-up, the median number of participants lost to follow-up was 6 (IQR, 1–13). 12 (40%) trials did not report the number of lost to follow-up, 6 (20%) trials had a number of lost to follow-up less than 10% of their respective total sample size, 10 studies (33.3%) had a number of lost to follow-up accounted for 10-20% of their respective total sample size, and in 2 studies, the number of lost to follow-up was more than 20% of the total sample size. According to the quality assessment of included RCTs, 9 trials were at low risk of bias, 2 were at high risk and the remaining 19 at some concerns of risk of bias. A summary of the RoB 2.0 results is shown in Table 2.

**Table 1:** Trial characteristics

Study ID	Funding	Blinding status	Analysis principle	Pathological type	TCM differentiation	Treatment flexibility	Type of control	Outcome	Sample size (M/F)		Lost to follow-up	FI*
									T	C		
Wang 2006 [30]	Yes	Double	ITT	IBS-D	Yes	No	placebo	③	13/16	18/10	NR	5
G.Cappello 2007 [31]	Unclear	Double	ITT	IBS-M	No	No	placebo	②	6/18	6/20	7	2
Zhang 2009 [32]	Yes	Unclear	ITT	IBS-D	Yes	Yes	standard	①	30/23	35/19	NR	2
Yuri 2009 [33]	Yes	Double	ITT	IBS-M	No	No	placebo	②	5/30	5/30	10	1
Li 2010 [34]	Yes	Double	ITT/PP	IBS-D	Yes	No	placebo	①	16/14	16/14	1	1
Zhang 2010 [35]	Yes	Assessor	PP	IBS-D	Yes	Yes	standard	①	103/77	94/86	8	8
Tang 2011 [36]	Unclear	Double	ITT/PP	IBS-D	No	No	placebo	⑤	19/9	18/12	2	7
Zhang 2013 [37]	Unclear	Unclear	ITT	IBS-D	Yes	No	standard	①	16/14	15/15	NR	1
Fu 2013 [38]	Yes	Unclear	ITT	IBS-D	Yes	No	standard	①	22/46	24/44	NR	2
Li 2014 [39]	Yes	Unclear	ITT	IBS-D	Yes	Yes	standard	①	28/25	30/23	NR	2
Piero 2014 [40]	Unclear	Double	ITT	IBS-M	No	No	placebo	③	17/41	22/36	NR	4
Liu 2014 [41]	Unclear	Unclear	ITT	IBS-D	No	No	standard	①	48/52	51/49	NR	11
Hu 2015 [42]	Yes	Unclear	ITT	IBS-D	Yes	No	standard	①	28/42	31/39	NR	4
Cheng 2015 [43]	Unclear	Unclear	PP	IBS-D	Yes	No	standard	①	15/17	14/16	2	1
Felix 2017 [44]	Yes	Double	ITT	IBS-M	No	No	placebo	②	12/31	15/32	7	9
M.Chen 2018 [45]	Yes	Double	ITT	IBS-D	Yes	No	placebo	⑤	41/39	31/49	5	3
Tang 2018 [46]	Unclear	Double	ITT/PP	IBS-D	No	No	placebo	①	62/37	66/41	10	13
Weng 2019 [47]	Yes	Unclear	PP	IBS-D	Yes	No	standard	①	16/21	18/19	6	1
Zeng 2019 [48]	Unclear	Unclear	PP	IBS-D	Yes	No	standard	①	23/9	20/13	5	1
Chen 2020 [49]	Yes	Unclear	ITT	IBS-D	Yes	No	standard	①	15/15	12/18	NR	1
Zhao 2020 [50]	Yes	Unclear	PP	IBS-D	Yes	No	standard	④	22/11	19/14	6	1
Zheng 2020 [51]	Yes	Unclear	PP	IBS-C	Yes	Yes	as usual	①	28/44	30/42	13	1
Ghasem 2020 [52]	Yes	Double	ITT	IBS-M	No	No	placebo	③	13/19	12/20	4	8
Yu 2020 [53]	Unclear	Unclear	PP	IBS-C	Yes	No	standard	①	12/20	7/25	6	1
Li 2021 [54]	Unclear	Unclear	ITT	IBS-C	No	No	standard	④	13/12	14/11	NR	1
Kong 2021 [55]	Unclear	Unclear	ITT	IBS-C	Yes	No	standard	①	16/14	18/12	NR	1
Jin 2021 [56]	Unclear	Unclear	ITT/PP	IBS-D	Yes	No	standard	①	36/30	35/32	7	11
Guo 2022 [57]	Yes	Double	PP	IBS-D	Yes	No	placebo	④	24/34	22/36	9	1
Zou 2022 [58]	Yes	Unclear	ITT	IBS-D	Yes	Yes	standard	①	12/19	14/17	4	1
Wang 2022 [59]	Yes	Unclear	ITT	IBS-D	Yes	No	standard	③	47/37	45/39	NR	1

*Abbreviation* NR, not reported. M, male; F, female; T, treatment group; C, control group. IBS-D, diarrhea-predominant irritable bowel syndrome; IBS-C, constipation-predominant irritable bowel syndrome; IBS-M, mixed predominant irritable bowel syndrome

①Effective rate; ②Response rate on IBS-SSS Scale; ③Response rate to abdominal pain; ④Response rate on Bristol Stool Scale; ⑤Adequate relief

*Fragility Index was calculated from a 2 × 2 contingency table by gradually changing one patient at a time from “event” status to “non-event” status in the experimental group (defined as the group with a high number of events in a positive study) until the bilateral Fisher precision tests did not generate statistical significance. The larger the Fragility Index, the better is the robustness of the RCT

**Table 2:** Review of author’s judgements about each risk of bias item presented as percentages across all included studies

	Randomization process, *n* (%)	Deviations from intended interventions, *n* (%)	Missing outcome data, *n* (%)	Measurement of the outcome, *n* (%)	Selection of the reported result, *n* (%)	Overall, *n* (%)
Low risk	21 (70)	11 (36.67)	25 (83.33)	10 (33.33)	30 (100.00)	9 (30.00)
Some concerns	9 (30)	17 (56.67)	5 (16.67)	20 (66.67)	0 (0)	19 (63.33)
High risk	0 (0)	2 (6.67)	0 (0)	0 (0)	0 (0)	2 (6.67)

### Fragility index

**Fig. 3:**
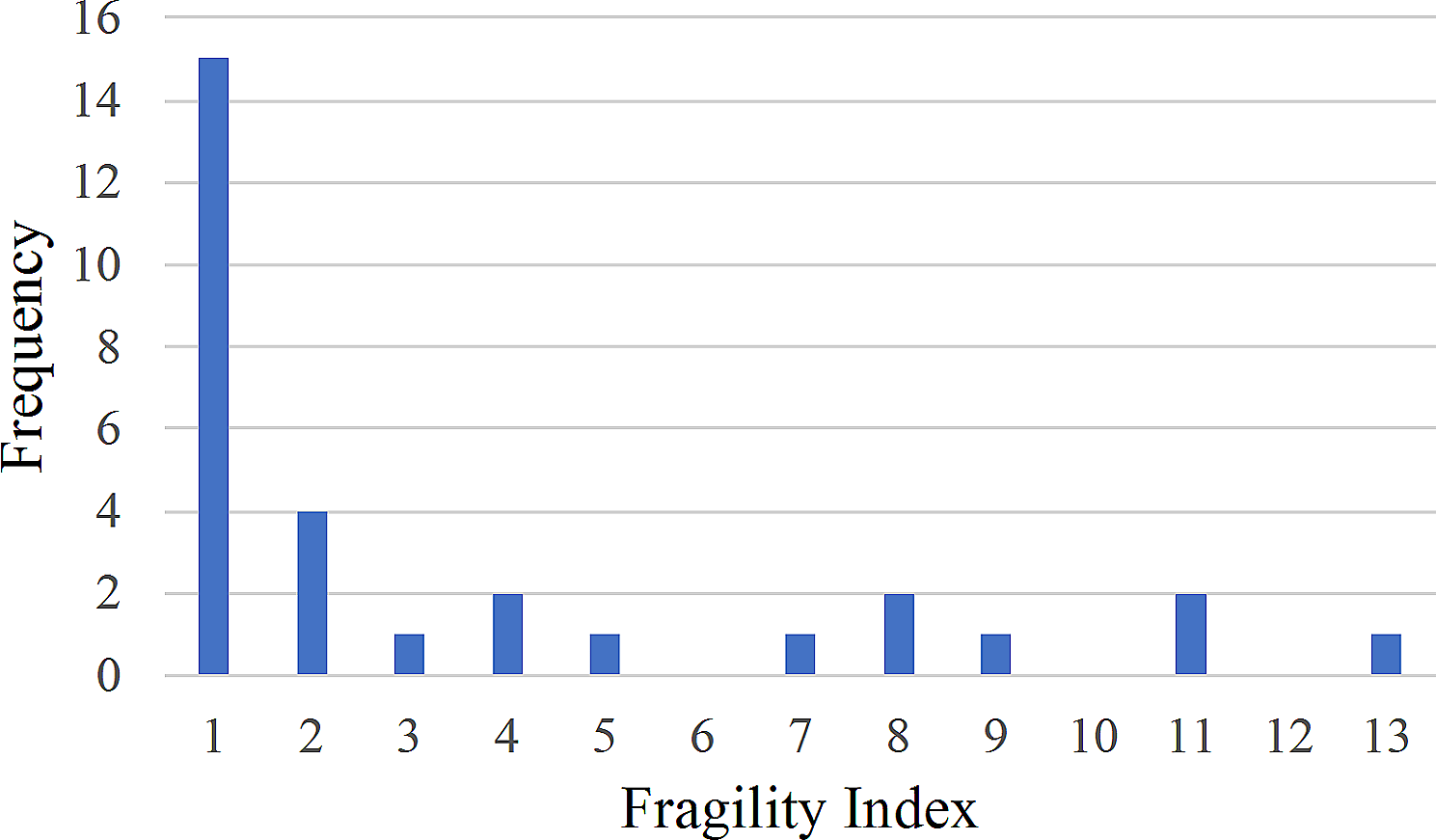
Distribution of fragility index for all trials

The overall median FI was found to be 1.5 (IQR, 1–5). Among the trials, half of them (*n* = 15, 50%) had an FI of 1, indicating that even a small change of one patient in the treatment group from an event to a non-event could lead to a loss of statistical significance in the RCT findings, raising concerns about the robustness and reliability of the findings. Meanwhile, in only 3 trials (10%), the FI exceeded 10, suggesting a higher robustness of their results to alterations. Notably, the total number lost to follow-up exceeded the FI in 12 (66.7%) trials, which might have a significant impact on the robustness of their outcomes (Fig. 3).

### Associations between the fragility index and study characteristics

Table 3 shows the results of the linear regression analysis. It was observed that larger total sample size (β, 0.27; 95% CI 0.09, 0.46; *P* = 0.005), low risk of overall bias (β, -2.15; 95% CI -3.96, -0.34; *P* = 0.022), and larger numbers of events (β, 0.22; 95% CI 0.02, 0.45; *P* = 0.043) were associated with more robust results. Furthermore, in the different domains of the Cochrane’s RoB 2.0 tool, we explored potential positive influence of low risk assessment of randomization process (β, 2.78; 95% CI 0.77, 5.07; *P* = 0.010), deviations from intended interventions (β, 2.92; 95% CI 0.86, 4.90; *P* = 0.007), and measurement of the outcome (β, 3.26; 95% CI 1.19, 5.21; *P* = 0.003) on FI. Of interest, trials without adopting TCM syndrome differentiation for inclusion criteria of the patients were associated with more robust results (β, 2.32; 95% CI 0.06, 4.57; *P* = 0.044). However, the linear regression did not identify any significant differences in the FI concerning positive funding support, the flexibility of the intervention treatment tailored to the patient’s condition, the pathological type of IBS, the proportion of lost to follow-up or the type of control intervention.

**Table 3:** Association between trial characteristics and the fragility index using linear regression

Variable	β-coefficient^a^ (95% CI)	*P*-value
Total sample size	0.27 (0.09–0.46)	0.005
Number of events	0.22 (0.02–0.45)	0.043
Funding	1.61 (-1.17–4.39)	0.246
**TCM syndrome differentiation**		
Yes	Referent	
No	2.32 (0.06–4.57)	0.044
**Flexibility of interventional treatment**		
Yes	Referent	
No	0.88 (− 2.86–4.62)	0.633
**Pathological type**		
Mixed	Referent	
Diarrhea	-1.08 (-4.79–2.62)	0.554
Constipation	-3.8 (-8.81–1.21)	0.132
**Type of control intervention**		
Non-placebo	Referent	
Placebo	2.17 (-0.61–4.49)	0.120
**Lost to follow-up**		
≤ 10%	Referent	
10 − 20%	-1.09 (-4.89–1.49)	0.284
20 − 30%	-0.81 (-7.05–3.05)	0.423
Not reported	-0.89 (-4.42–1.76)	0.383
**Overall risk of bias**		
Some concerns/high risk	Referent	
Low risk	2.15 (0.34–3.96)	0.022
**Risk of randomization process**		
Some concerns/high risk	Referent	
Low risk	2.78 (0.77–5.07)	0.010
**Risk of deviations from intended interventions**		
Some concerns/high risk	Referent	
Low risk	2.92 (0.86–4.90)	0.007
**Risk of missing outcome data**		
Some concerns/high risk	Referent	
Low risk	0.55 (-2.17- 3.77)	0.585
**Risk of measurement of the outcome**		
Some concerns/high risk	Referent	
Low risk	3.26 (1.19–5.21)	0.003

*Abbreviation* CI, confidence interval

^a^The β-coefficient refers to the FI difference, compared to a trial without this feature or compared to a referent group

## Discussion

### Summary of findings

The median FI was 1.5 (IQR, 1–5) among included RCTs reported CHM treatment on IBS which conducted a statistically significant result, indicating that even a small change of one or two patients in the treatment arm from a positive target outcome event to a negative target outcome event could lead to a loss of statistical significance in the RCT findings. Furthermore, in 40% of the trials, the number of patients lost to follow-up exceeded the respective FI. The study also identified that increased FI was significantly associated with no TCM syndrome differentiation for inclusion criteria of the patients, larger total sample size, low risk of bias, and larger numbers of events, but was not associated with whether there was a funding support or not, the pathological type of IBS, the type of control intervention, the proportion of lost to follow-up or the individualized treatments re patients’ condition.

### Strengths and limitations

The present study stands as the first to report the FI of RCTs comparing different formulations of CHM for treating IBS. The FI provides a straightforward measure, represented by the number of individual patients, that can assist clinical practitioners, patients, and policymakers in assessing the strength of research conclusions. We conducted a comprehensive search of the literature and explored the association between the FI and the sample size, the number of events, the funding support, the pathological type of IBS, the type of intervention comparisons, the proportion of lost to follow-up, and result of the risk of bias assessment. Furthermore, we also considered the unique characteristics of RCTs in TCM such as whether trials adopted TCM syndrome differentiation for inclusion criteria of the patients and whether there were individualized treatments according to patients’ symptoms. As limitations, the regression analyses were univariable since the small sample size that included in this study and a high degree of multicollinearity that may lead to a loss of statistical significance of analyses and potentially excluding important variables from the analyses limited the conduction of multivariable analyses, such as the set of sample size and number of events as a larger sample size leads to a larger number of events, and the set of risk of randomization process, risk of deviations from intended interventions, and risk of measurement of the outcome as they were various domains of one tool. Furthermore, the eligible criteria for outcomes were limited to internationally recognized measure, which excluded some outcome measures typically associated with TCM syndromes, such as the effective rate measured by self-made TCM symptom scales as these outcome measurements lacked certain reliability and validity, which may have implications on the overall assessment of trial robustness.

### Relationship with previous studies

Our findings align with previously reported FI scores in various medical and surgical fields, such as peri-operative care (median, 2; IQR, 1–3) [60], otolaryngology (median, 3; IQR, 1-7.5) [61], anesthesia and critical care (median, 2; IQR, 1-3.5) [12], and emergency medicine (median, 4; IQR, 2–10) [62]. In contrast, common solid tumor trials had a higher median FI of 28 (range, 2–322) [13]. This discrepancy in trial robustness may be attributed to the number of events in the trials. Among the 30 trials included in our study, the number of events was notably smaller when compared to the FI review of anti-cancer drugs for solid tumors, where the median number of events was 4 versus 336.5. A larger number of events tends to be positively correlated with a higher FI score, a trend observed consistently in both our study and previous research. Apart from being influenced by the number of events, the FI is inherently linked to the sample size. Of interest, our findings are consistent with the previous results, of which the majority have shown a positive association between the FI and sample size [10, 11].

Few of the published studies on FI have analyzed the results from the perspective of study quality evaluation. Only one study on hand surgery [11] evaluated the risk of bias of the included trials, but since the study included only five eligible studies, the author did not analysis its relationship with FI. Consistent with our expectation, low risk assessment of bias especially in the domain of randomization process, deviations from intended interventions, and measurement of the outcome were positively associated with more robustness results. For RCTs with a higher risk of bias, the probability of generating misleading results exceeds 50%, even if the results are statistically significant. This highlights the importance of conducting rigorous study designs in randomization, avoiding bias through stringently monitored, and blinding outcome assessors as could as possible to detect meaningful effects. Additionally, the replication of findings in independent studies can further strengthen the validity and reliability of research outcomes.

In more than half the RCTs that clearly reported lost to follow-up, more participants were lost to follow-up than would be required to make the result nonsignificant based on the corresponding trial’s FI. The number of lost to follow-up was expected to have an influence on the robustness of result as the more patients lost to follow-up, the more outcomes would be missed or biased by the inadequate treatment and the result of patients who lost to follow-up might be negative lead to a smaller FI. When all participants in the study were considered, the statistical significance of the results may be reversed, raising concerns about the robustness and reliability of the findings. A previous study which reviewed the FI of 399 RCTs found that the feature of not reported the number of lost to follow-up in the results in RCTs was a significantly associated with larger FI^28^. However, the linear regression did not identify any significant difference in the FI on the proportion of lost to follow-up in this study. Considering the small sample size of included studies and there were over 1/3 RCTs that did not report the number of lost to follow-up, the nonsignificant association in this study may be influenced by inadequate methodological reported limitations.

### Implications

Treatment decisions often start with the decision of whether a treatment effect is believed to exist. The FI serves as a significant supplementary indicator for *P* values that may assist clinicians in determining the confidence they should have in the result [63]. A fragile result of patient’s concerned outcome may influence clinicians to draw appropriate inferences regarding the low confidence in the effect of a specific CHM treatment effect for IBS. Presenting both *P* value and FI for a trial outcome can help clinicians to better understand not only the statistical significance of the outcome but also the fragility, that is sensitivity of the positive outcome to small changes of the number of positive events and missing data. The significance of fragility is emphasized by the proportion of RCTs that initially presented statistically significant results but were subsequently found to be either unsuccessful (16%) or demonstrated effects that were considerably lower than previously reported (16%) [64]. While a low FI indicates a fragile trial result, solutions that increase the robustness of trial result may be adequate sample size based on proper calculation, scientifically rigorous study design, and proper control of confounding factors. When fragile results are derived from low-quality RCTs, other clinical trial designs, such as objective performance criteria based single-armed trial, are worth exploring.

Interestingly in this study, there was a significant relationship between the inclusion criteria of patients and whether there was a TCM syndrome differentiation and the FI suggested that studies that implemented no TCM syndrome differentiation inclusion criteria tended to have more robust results. Although there were clear criteria in the included trials for the syndrome diagnosis of disease, no trials mentioned rigorous training and quality control for the diagnosis of the individualized syndrome, and it was unclear whether the accuracy and consistency of the identification of the syndrome were examined uniformly across different researchers before the start of the trial. A previous study [65] has shown that there was a low degree of consistency in TCM diagnoses and individualized prescriptions among TCM doctors even with the same qualifications (diagnosis: kappa = 31.7%, prescription: kappa = 35.0%). The inconsistent “TCM diagnosis” results may make trials more fragile. A detailed standard of practice “TCM diagnosis” and quality control calibration for consistency of TCM diagnostic results among clinicians before the implement of trial could be a potential strategy to address this issue.

TCM interventions need to adapt to the dynamic syndromes [66]. In clinical trials, such individualized treatment brings higher heterogeneity to the interventions [67, 68], which may increase difficulty of trial management. However, our findings demonstrated that there was no difference in the FI concerning whether personalized prescriptions were modified according to each patient’s specific symptoms or not. Not only TCM but also western conventional medicine applies individualized treatments to some extent even in a RCT with rigorous requirement for interventions due to medical needs. Our result provides evidence to relieve concern to the potential adverse impact of individualized treatment to the robustness of trial results. However, the relatively small amount of literature that included features of individualized interventions (*n* = 5, 16.67%) may lead to false-negative results. Besides, the lack of association between funding support and FI indicated that the robustness of trial was not affected by commercial conflicts of interest and highlighted the importance of the role that a rigorous protocol design play in the robustness of RCT.

## Conclusion

The majority of CHM IBS RCTs with positive results were found to be fragile. Ensuring adequate sample size and low risk of bias should be addressed to increase the robustness of the RCTs. We recommend reporting the FI as one of the components of sensitivity analysis in future RCTs to facilitate the assessment of the fragility of trials.

### Electronic supplementary material

Below is the link to the electronic supplementary material.

Supplementary Material 1

## Data Availability

The data that support the findings of this study are available from the corresponding author, (Yutong Fei [E-mail: feiyt@bucm.edu.cn]), upon reasonable request.

## Declarations

### Provenance and peer review

Not commissioned; externally peer reviewed.
