# Supplementary material for: How fragile the positive results of Chinese herbal medicine randomized controlled trials on irritable bowel syndrome are?
Source: BMC Complement Med Ther. 2024 Aug 14;24:300. doi: 10.1186/s12906-024-04561-8 (PMC11323352; doi:10.1186/s12906-024-04561-8)
Supplement: Supplementary file 1 — Supplementary Material 1 [file 12906_2024_4561_MOESM1_ESM.docx]

**Supplementary Material**

**How Fragile about the Results of Randomized Controlled Trial on Irritable Bowel Syndrome**

[Table S1. Full search strategy 2](#_Toc151474781)

[Table S2. PRISMA 2020 Checklist of information to include when reporting a systemtic review 9](#_Toc151474782)

# Table S1. Full search strategy

| Database | Search Strategy |
| --- | --- |
| MEDLINE (Ovid) | 1 exp drugs, Chinese herbal/ 2 exp alternative medicine/ 3 exp plant extracts/ 4 exp plants, medicinal/ 5 exp herbal medicine/ 6 exp drugs, non-prescription/ or exp plant preparations/ 7 medicine, Chinese traditional.sh. 8 Phytotherapy/ 9 exp east asian traditional/ 10 exp medicine, Chinese traditional/  11 exp medicine, kampo/  12 exp medicine, korean traditional/ 13 exp medicine, tibetan traditional/ 14 exp medicine, mongolian traditional/ 15 exp shamanism/ 16 (TCM or CHM).ti,ab,kw. 17 "herb*".ti,ab,kw. 18 ((Chinese or China or tranditional or oriental or alternative or Complementary) adj3 (medicin* or drug* or medication* or formula*)).ti,ab,kw. 19 (plant or plants or flower* or seeds or seed or tea or teas or tree or trees or phytotherap* or phyto-therap* or phyto-drug* or phytodrug* or beverage* or oil* or extract* or phytopharmaceutical or phytopharmaceutical or phytopharmaceutical).ti,ab,kw. 20 1 or 2 or 3 or 4 or 5 or 6 or 7 or 8 or 9 or 10 or 11 or 12 or 13 or 14 or 15 or 16 or 17 or 18 or 19  21 exp irritable bowel syndrome/ 22 exp colonic diseases, functional/ 23 ((irritable or spastic or unstable or spasm) adj (colon* or bowel)).ti,ab,kw. 24 IBS.ti,ab,kw. 25 functional abdominal pain.ti,ab,kw. 26 functional gastrointestinal disorders.ti,ab,kw. 27 mucous colitis.ti,ab,kw. 28 21 or 22 or 23 or 24 or 25 or 26 or 27 29 recurrent.ti,ab,kw. 30 chronic.ti,ab,kw.  31 excessive.ti,ab,kw. 32 hypersensitivity.ti,ab,kw. 33 29 or 30 or 31 or 32 34 "diarrhea*".ti,ab,kw. 35 "diarrhoe*".ti,ab,kw. 36 "diarhe*".ti,ab,kw. 37 "diarhoe*".ti,ab,kw. 38 gastroenteritis.ti,ab,kw. 39 abdominal pain.ti,ab,kw. 40 abdominal cramp.ti,ab,kw. 41 bloating.ti,ab,kw. 42 disturbed defecation.ti,ab,kw. 43 "constipat*".ti,ab,kw. 44 "flatulen*".ti,ab,kw. 45 34 or 35 or 36 or 37 or 38 or 39 or 40 or 41 or 42 or 43 or 44 46 33 and 45 47 28 or 46 48 randomized controlled trial.pt. 49 controlled clinical trial.pt. 50 "random*".ab,ti,kw. 51 groups.ab,ti,kw. 52 placebo.ab,ti,kw. 53 RCT.ti,ab,kw. 54 prospective study.ti,ab,kw. 55 (clin* adj25 trial*).ti,ab,kw.  56 ((singl* or doubl* or treb* or tripl*) adj25 (blind* or mask*)).ti,ab,kw. 57 clinical trials as topic.sh. 58 drug therapy.ti,ab,kw. 59 trial.ti,ab,kw. 60 48 or 49 or 50 or 51 or 52 or 53 or 54 or 55 or 56 or 57 or 58 or 59 61 20 and 47 and 60 |
| EMBASE | ('drugs, chinese herbal'/exp OR 'drugs, chinese herbal' OR 'alternative medicine'/exp OR 'plant extracts'/exp OR 'plants, medicinal'/exp OR 'drugs, non-prescription'/exp OR 'plant preparations'/exp OR phytotherapy OR 'east asian traditional' OR 'medicine, kampo'/exp OR 'medicine, chinese traditional'/exp OR 'medicine, korean traditional'/exp OR 'medicine, tibetan traditional'/exp OR 'medicine, mongolian traditional'/exp OR 'shamanism'/exp OR 'chinese medicine'/exp OR tcm:ab,kw,ti OR chm:ab,kw,ti OR herb*:ab,kw,ti OR (((chinese OR china OR tranditional OR oriental OR alternative OR complementary) NEAR/3 (medicin* OR drug* OR medication* OR formula*)):ab,kw,ti) OR 'herbal medicine'/exp OR 'plant medicinal product'/exp OR plant:ab,kw,ti OR plants:ab,kw,ti OR flower*:ab,kw,ti OR seeds:ab,kw,ti OR seed:ab,kw,ti OR tea:ab,kw,ti OR teas:ab,kw,ti OR tree:ab,kw,ti OR trees:ab,kw,ti OR phytotherap*:ab,kw,ti OR 'phyto therap*':ab,kw,ti OR 'phyto drug*':ab,kw,ti OR phytodrug*:ab,kw,ti OR beverage*:ab,kw,ti OR oil*:ab,kw,ti OR extract*:ab,kw,ti OR phytopharmaceutical:ab,kw,ti OR 'phytotherapy'/exp) AND ('irritable bowel syndrome'/exp OR 'irritable bowel syndrome' OR 'colonic diseases, functional'/exp OR (((irritable OR spastic OR unstable OR spasm) NEAR/2 (colon* OR bowel)):ti,ab,kw) OR ibs:ab,kw,ti OR 'functional abdominal pain':ab,kw,ti OR 'functional gastrointestinal disorders':ab,kw,ti OR 'mucous colitis':ab,kw,ti OR (('excessive':ab,kw,ti OR 'hypersensitivity':ab,kw,ti OR 'chronic':ab,kw,ti OR 'recurrent':ab,kw,ti) AND ('diarrhea*':ab,kw,ti OR 'diarrhoe*':ab,kw,ti OR 'diarhe':ab,kw,ti OR 'diarhoe*':ab,kw,ti OR 'gastroenteritis':ab,kw,ti OR 'abdominal pain':ab,kw,ti OR 'abdominal cramp':ab,kw,ti OR 'bloating':ab,kw,ti OR 'disturbed defecation':ab,kw,ti OR 'constipat*':ab,kw,ti OR 'flatulen*':ab,kw,ti))) AND ('randomized controlled trial'/exp OR 'controlled clinical trial'/exp OR 'clinical trial'/exp OR randomized:ab,kw,ti OR randomly:ab,kw,ti OR random*:ab,kw,ti OR groups:ab,kw,ti OR rct:ab,kw,ti OR 'prospective study':ab,kw,ti OR ((clin* NEAR/25 trial*):ab,kw,ti) OR (((singl* OR doubl* OR treb* OR tripl*) NEAR/25 (blind* OR mask*)):ab,kw,ti) OR placebo:ab,kw,ti) |
| Web of Science | #1 (((((((((((((((((((TS=(drugs, Chinese herbal)) OR TS=(alternative medicine)) OR TS=(plant extracts)) OR TS=(plants, medicinal)) OR TS=(herbal medicine)) OR TS=(drugs, non-prescription)) OR TS=( plant preparations)) OR TS=(medicine, Chinese traditional)) OR TS=(Phytotherapy)) OR TS=(east asian traditional)) OR TS=(medicine, Chinese traditional)) OR TS=(medicine, kampo)) OR TS=(medicine, korean traditional)) OR TS=(medicine, tibetan traditional)) OR TS=(medicine, mongolian traditional)) OR TS=(shamanism)) OR TS=((TCM or CHM))) OR TS=(herb*)) OR TS=(((chinese or china or tranditional or oriental or alternative or Complementary) adj3 (medicin* or drug* or medication* or formula*)))) OR TS=((plant or plants or flower* or seeds or seed or tea or teas or tree or trees or phytotherap* or phyto-therap* or phyto-drug* or phytodrug* or beverage* or oil* or extract* or phytopharmaceutical or phytopharmaceutical or phytopharmaceutical)) #2 ((((((TS=(irritable bowel syndrome)) OR TS=(colonic diseases, functional)) OR TS=(((irritable or spastic or unstable or spasm) adj (colon* or bowel)))) OR TS=(IBS)) OR TS=(functional abdominal pain)) OR TS=(functional gastrointestinal disorders)) OR TS=(mucous colitis) #3 (((TS=(recurrent)) OR TS=(chronic)) OR TS=(excessive)) OR TS=(hypersensitivity) #4 ((((((((((TS=(diarrhea*)) OR TS=(diarrhoe*)) OR TS=(diarhe*)) OR TS=(diarhoe*)) OR TS=(gastroenteritis)) OR TS=(abdominal pain)) OR TS=(abdominal cramp)) OR TS=(bloating)) OR TS=(disturbed defecation)) OR TS=(constipat*)) OR TS=(flatulen*) #5 #3 and #4 #6 #2 or #5 #7 (((((((((((TS=(randomized controlled trial)) OR TS=(controlled clinical trial)) OR TS=(clinical trial)) OR TS=(randomized)) OR TS=(randomly)) OR TS=(random*)) OR TS=(groups)) OR TS=(RCT)) OR TS=(prospective study)) OR TS=((clin* adj25 trial*))) OR TS=(((singl* or doubl* or treb* or tripl*) adj25 (blind* or mask*)))) OR TS=(placebo) #8 #7 AND #6 AND #1 |
| Cochrane library | #1 MeSH descriptor: [Medicine, Chinese Traditional] explode all trees #2 (chinese or china or tranditional or oriental or alternative or Complementary) near (medicin* or drug* or medication* or formula*):ti,ab,kw #3 (TCM or TCHM):ti,ab,kw #7 MeSH descriptor: [Herbal Medicine] explode all trees #8 (herb*):ti,ab,kw #9 MeSH descriptor: [Plant Preparations] explode all trees #10 (plant or plants or flower* or seeds or seed or tea or teas or tree or trees or phytotherap* or phyto-therap* or phyto-drug* or phytodrug* or beverage* or oil* or extract* or phytopharmaceutical or phytopharmaceutical phytopharmaceutical)ti,ab,kw #11 ((chinese or china or tranditional or oriental or alternative or Complementary) near (medicin* or drug* or medication* or formula*)):ti,ab,kw #12 MeSH descriptor: [Phytotherapy] explode all trees #13 MeSH descriptor: [Nonprescription Drugs] explode all trees #14 MeSH descriptor: [Plant Preparations] explode all trees #15 #1 or #2 or #3 or #4 or #5 or #6 or #7 or #8 or #9 or #10 or #11 or #12 or #13 or #14  #16 MeSH descriptor: [Colonic Diseases, Functional] explode all trees #17 MeSH descriptor: [Irritable Bowel Syndrome] explode all trees #18 ((irritable or spastic or unstable or spasm) near (colon* or bowel)):ti,ab,kw #19 IBS:ti,ab,kw #20 functional abdominal pain:ti,ab,kw #21 (mucous colitis):ti,ab,kw #22 functional abdominal pain:ti,ab,kw #23 functional gastrointestinal disorders:ti,ab,kw #24 #16 or #17 or #18 or #19 or #20 or #21 or #22 or #23 #25 recurrent:ti,ab,kw #26 chronic:ti,ab,kw #27 excessive:ti,ab,kw #28 hypersensitivity:ti,ab,kw #29 #25 or #26 or #27 or #28 #30 "diarrhea*":ti,ab,kw #31 "diarrhoe*":ti,ab,kw #32 "diarhe*":ti,ab,kw #33 "diarhoe*":ti,ab,kw #34 gastroenteritis:ti,ab,kw #35 "abdominal pain":ti,ab,kw #36 "abdominal cramp":ti,ab,kw #37 bloating:ti,ab,kw #38 "disturbed defecation":ti,ab,kw #39 "constipat*":ti,ab,kw #40 "flatulen*":ti,ab,kw #41 #30 or #31 or #32 or #33 or #34 or #35 or #36 or #37 or #38 or #39 or #40 #42 #29 and #41 #43 #24 or #42  #44 randomized controlled trial:pt #45 controlled clinical trial:pt #46 "random*":ab,ti,kw #47 groups:ab,ti,kw #48 placebo:ab,ti,kw #49 RCT:ti,ab,kw #50 prospective study:ti,ab,kw #51 (clin* near 25 trial*):ti,ab,kw #52 ((singl* or doubl* or treb* or tripl*) near (blind* or mask*)):ti,ab,kw #53 drug therapy:ti,ab,kw #54 trial:ti,ab,kw #55 MeSH descriptor: [Clinical Trials as Topic] explode all trees #56 #44 or #45 or #46 or #47 or #48 or #49 or #50 or #51 or #52 or #53 or #54 or #55 #57 #15 and #43 and #56 |
| China National Knowledge Infrastructure (CNKI) | SU=('肠易激综合征'+'IBS'+'肠*激惹'+'肠易激*') AND SU=('中药'+'中医药'+'草药'+'中成药'+'中医'+'中西医'+'辨证论治'+'方剂'+'复方'+'本草'+'生药'+'草本药'+'植物药'+'饮片'+'民族药'+'民间方'+'汤'+'方'+'法'+'散'+'膏') AND FT=('随机'+'RCT') |
| Wan-Fang Databases | (主题:("肠易激综合征" or "IBS" or "肠*激惹" or "肠易激*") and 主题:("中药" or "中医药" or "中医" or "中西医" or "辨证论治" or "中成药" or "草药" or "方剂" or "复方" or "本草" or "生药" or "草本药" or "植物药" or "饮片" or "汤" or "方" or "法" or "散" or "膏" or "民族药" or "民间方") and 全部:("随机" or "RCT")) |
| Chinese Scientific Journal Database | (((M="肠易激综合征" OR "IBS" OR "肠*激惹" OR "肠易激*") OR (R="肠易激综合征" OR "IBS" OR "肠*激惹" OR "肠易激*")) AND (M=中药 OR 中医药 OR 中医 OR 中西医 OR 辨证论治 OR 中成药 OR 草药 OR 方剂 OR 复方 OR 本草 OR 生药 OR 草本药 OR 植物药 OR 饮片 OR 汤 OR 方 OR 法 OR 散 OR 膏 OR 民族药 OR 民间方)) AND (U=随机 OR RCT) |
| Chinese BidMedicine (SinoMed) | ((((( "中药"[常用字段:智能] OR "中医药"[常用字段:智能] OR "中医"[常用字段:智能] OR "中西医"[常用字段:智能] OR "辨证论治"[常用字段:智能] OR "中成药"[常用字段:智能] OR "草药"[常用字段:智能] OR "方剂"[常用字段:智能] OR "复方"[常用字段:智能] OR "本草"[常用字段:智能] OR "生药"[常用字段:智能] OR "草本药"[常用字段:智能] OR "植物药"[常用字段:智能] OR "饮片OR 汤"[常用字段:智能] OR "方"[常用字段:智能] OR "法"[常用字段:智能] OR "散"[常用字段:智能] OR "膏"[常用字段:智能] OR "民族药"[常用字段:智能] OR "民间方"[常用字段:智能]))) AND ("随机"[全部字段:智能] OR "RCT"[常用字段:智能]))) AND (( "肠易激综合征"[常用字段:智能] OR " IBS "[常用字段:智能] OR "肠*激惹"[常用字段:智能] OR "肠易激*"[常用字段:智能])) |

# Table S2. PRISMA 2020 Checklist of information to include when reporting a systemtic review

| **Section and Topic** | **Item #** | **Checklist item** | **Location where item is reported** |
| --- | --- | --- | --- |
| **TITLE** | | |  |
| Title | 1 | Identify the report as a systematic review. | 1 |
| **ABSTRACT** | | | 2 |
| Abstract | 2 | See the PRISMA 2020 for Abstracts checklist. |  |
| **INTRODUCTION** | | |  |
| Rationale | 3 | Describe the rationale for the review in the context of existing knowledge. | 3 |
| Objectives | 4 | Provide an explicit statement of the objective(s) or question(s) the review addresses. | 4 |
| **METHODS** | | |  |
| Eligibility criteria | 5 | Specify the inclusion and exclusion criteria for the review and how studies were grouped for the syntheses. | 5-6 |
| Information sources | 6 | Specify all databases, registers, websites, organisations, reference lists and other sources searched or consulted to identify studies. Specify the date when each source was last searched or consulted. | 4 |
| Search strategy | 7 | Present the full search strategies for all databases, registers and websites, including any filters and limits used. | 4  Table S1 in Supplemental Material |
| Selection process | 8 | Specify the methods used to decide whether a study met the inclusion criteria of the review, including how many reviewers screened each record and each report retrieved, whether they worked independently, and if applicable, details of automation tools used in the process. | 6 |
| Data collection process | 9 | Specify the methods used to collect data from reports, including how many reviewers collected data from each report, whether they worked independently, any processes for obtaining or confirming data from study investigators, and if applicable, details of automation tools used in the process. | 6-7 |
| Data items | 10a | List and define all outcomes for which data were sought. Specify whether all results that were compatible with each outcome domain in each study were sought (e.g. for all measures, time points, analyses), and if not, the methods used to decide which results to collect. | 6 |
|  | 10b | List and define all other variables for which data were sought (e.g. participant and intervention characteristics, funding sources). Describe any assumptions made about any missing or unclear information. | 6 |
| Study risk of bias assessment | 11 | Specify the methods used to assess risk of bias in the included studies, including details of the tool(s) used, how many reviewers assessed each study and whether they worked independently, and if applicable, details of automation tools used in the process. | Not applicable |
| Effect measures | 12 | Specify for each outcome the effect measure(s) (e.g. risk ratio, mean difference) used in the synthesis or presentation of results. | 7 |
| Synthesis methods   | 13a | Describe the processes used to decide which studies were eligible for each synthesis (e.g. tabulating the study intervention characteristics and comparing against the planned groups for each synthesis (item #5)). | 7 |
|  | 13b | Describe any methods required to prepare the data for presentation or synthesis, such as handling of missing summary statistics, or data conversions. | 7 |
|  | 13c | Describe any methods used to tabulate or visually display results of individual studies and syntheses. | 7 |
|  | 13d | Describe any methods used to synthesize results and provide a rationale for the choice(s). If meta-analysis was performed, describe the model(s), method(s) to identify the presence and extent of statistical heterogeneity, and software package(s) used. | 7 |
|  | 13e | Describe any methods used to explore possible causes of heterogeneity among study results (e.g. subgroup analysis, meta-regression). | Not applicable |
|  | 13f | Describe any sensitivity analyses conducted to assess robustness of the synthesized results. | Not applicable |
| Reporting bias assessment | 14 | Describe any methods used to assess risk of bias due to missing results in a synthesis (arising from reporting biases). | Not applicable |
| Certainty assessment | 15 | Describe any methods used to assess certainty (or confidence) in the body of evidence for an outcome. | Not applicable |
| **RESULTS** | | |  |
| Study selection | 16a | Describe the results of the search and selection process, from the number of records identified in the search to the number of studies included in the review, ideally using a flow diagram. | 8 |
|  | 16b | Cite studies that might appear to meet the inclusion criteria, but which were excluded, and explain why they were excluded. | Not applicable |
| Study characteristics | 17 | Cite each included study and present its characteristics. | 8  Table 1 |
| Risk of bias in studies | 18 | Present assessments of risk of bias for each included study. | Not applicable |
| Results of individual studies | 19 | For all outcomes, present, for each study: (a) summary statistics for each group (where appropriate) and (b) an effect estimate and its precision (e.g. confidence/credible interval), ideally using structured tables or plots. | 9  Table 1, Table 2 |
| Results of syntheses | 20a | For each synthesis, briefly summarise the characteristics and risk of bias among contributing studies. | Not applicable |
|  | 20b | Present results of all statistical syntheses conducted. If meta-analysis was done, present for each the summary estimate and its precision (e.g. confidence/credible interval) and measures of statistical heterogeneity. If comparing groups, describe the direction of the effect. | 9 -10  Table 1, Table 2 |
|  | 20c | Present results of all investigations of possible causes of heterogeneity among study results. | Not applicable |
|  | 20d | Present results of all sensitivity analyses conducted to assess the robustness of the synthesized results. | Not applicable |
| Reporting biases | 21 | Present assessments of risk of bias due to missing results (arising from reporting biases) for each synthesis assessed. | Not applicable |
| Certainty of evidence | 22 | Present assessments of certainty (or confidence) in the body of evidence for each outcome assessed. | Not applicable |
| **DISCUSSION** | | |  |
| Discussion   | 23a | Provide a general interpretation of the results in the context of other evidence. | 10 |
|  | 23b | Discuss any limitations of the evidence included in the review. | 12 |
|  | 23c | Discuss any limitations of the review processes used. | 12 |
|  | 23d | Discuss implications of the results for practice, policy, and future research. | 13-14 |
| **OTHER INFORMATION** | | |  |
| Registration and protocol   | 24a | Provide registration information for the review, including register name and registration number, or state that the review was not registered. | 5 |
|  | 24b | Indicate where the review protocol can be accessed, or state that a protocol was not prepared. | 5 |
|  | 24c | Describe and explain any amendments to information provided at registration or in the protocol. | Not applicable |
| Support | 25 | Describe sources of financial or non-financial support for the review, and the role of the funders or sponsors in the review. | 15 |
| Competing interests | 26 | Declare any competing interests of review authors. | 15 |
| Availability of data, code and other materials | 27 | Report which of the following are publicly available and where they can be found: template data collection forms; data extracted from included studies; data used for all analyses; analytic code; any other materials used in the review. | 5 |
